# LoRaWAN Modeling and MCS Allocation to Satisfy Heterogeneous QoS Requirements

**DOI:** 10.3390/s19194204

**Published:** 2019-09-27

**Authors:** Dmitry Bankov, Evgeny Khorov, Andrey Lyakhov

**Affiliations:** 1 Wireless Networks Lab, Institute for Information Transmission Problems, Russian Academy of Sciences, 127051 Moscow, Russia; bankov@iitp.ru (D.B.); lyakhov@iitp.ru (A.L.); 2 Telecommunication Systems Lab, National Research University Higher School of Economics, 127051 Moscow, Russia

**Keywords:** internet of things, wireless sensor networks, LoRa, LoRaWAN, ALOHA, modeling, MCS allocation, performance evaluation

## Abstract

LoRaWAN infrastructure has become widely deployed to provide wireless communications for various sensor applications. These applications generate different traffic volumes and require different quality of service (QoS). The paper presents an accurate mathematical model of low-power data transmission in a LoRaWAN sensor network, which allows accurate validation of key QoS indices, such as network capacity and packet loss ratio. Since LoRaWAN networks operate in the unlicensed spectrum, the model takes into account transmission attempt failures caused by random noise in the channel. Given QoS requirements, we can use the model to study how the performance of a LoRaWAN network depends on the traffic load and other scenario parameters. Since in LoRaWAN networks the transmissions at different modulation and coding schemes (MCSs) typically do not collide, we use the model to assign MCSs to the devices to satisfy their QoS requirements.

## 1. Introduction

The LoRaWAN technology [1] is widely used for providing wireless communications for various internet of things (IoT) applications. The reasons for this are the open standardization procedure, the large number of companies involved in the ecosystem development process and the correctly chosen balance between the low complexity of the technology and low power consumption on the one side and its applicability to various use cases on the other side. Definitely, an essential peculiarity of LoRaWAN is that there is no need for compatibility with parent technologies, in contrast to NB-IoT [2] and Wi-Fi HaLow [3] which need to follow the ideology, the architecture and the protocols of Long Term Evolution (LTE) and Wi-Fi, respectively.

LoRa/LoRaWAN has a reliable physical layer (PHY) design that enables low-power long-range communications of sensor devices and resilience to noise. Although some MAC approaches used in LoRaWAN are oversimplified, which reduces the capacity of the network [4], the technology is flexible enough to apply to various use cases. Specifically, thanks to the acknowledged transmissions, LoRaWAN can be configured in a way to guarantee the delivery of the frames with a high probability. This feature enlarges its market from the geek scenarios where machine-type communication is just a cool option to the scenarios where it is a must-have feature for business processes: fire sensors, industrial and agriculture automation, remote control, e-health applications, etc. In such scenarios, various applications generate traffic that imposes different quality of service (QoS) requirements, namely the required delay, throughput, and the maximal affordable packet loss ratio (PLR).

Although LoRaWAN specification does not focus on QoS support, a LoRaWAN network can provide differentiated QoS. In the most widespread LoRaWAN networks, the only way to support QoS for the end-devices is to assign them to different modulation and coding schemes (MCS) properly. The assignment shall be done considering the fact that the MCS affects the duration of the transmitted data frames, the probability of their transmission being damaged by random noise or by the interference, and the impact of overlapping transmissions at different MCS on each other. At the same time, the MCS allocation algorithms for LoRaWAN are implementation-dependent, and the usage of straightforward approaches, such as the assignment of the fastest-possible MCS sometimes can be not the best solution, especially in the case of a heterogeneous network, when optimization of an average performance indicator, such as the throughput or PLR, does not necessarily result into all devices’ QoS requirements being satisfied.

In the paper, we present an accurate analytical model of LoRaWAN operation, which allows us to find out how the PLR and the data delivery time depend on the network configuration. The model also allows us to find the distribution of the delivery time, though such an index is less important for the majority of IoT applications. We also show how to use the model to configure a LoRaWAN network in order to satisfy different QoS requirements imposed by various IoT applications.

The model presented in this paper is an extension of our previous conference paper [5], where we have studied how the packet error rate (PER) depends on the load with and without capture effect. We extend the conference paper [5] with new methods to find delay distribution and packet loss ratio (PLR). Apart from the new key performance indices, we significantly modify the model and provide a detailed explanation. Specifically, we take into account transmission failures caused by random noise in the wireless channel and consider another distribution of distances between the motes and the gateway. Such changes help us to find that random noise and collisions have a different impact on network performance, e.g., PLR, even if PER is the same. Also, we significantly extend the numerical results by evaluating different scenarios and considering new key performance indices. Finally, we study the problem of the MCS selection—which has a strong practical value—and show how to use the model in the MCS selection process to satisfy heterogeneous QoS requirements of various traffic types in a LoRaWAN sensor network.

The rest of the paper is organized as follows. In Section 2, we explain the operation of the LoRaWAN network focusing on the features necessary for modeling. Section 3 gives a brief overview of the most relevant works. In Section 4, we describe in detail the considered scenario and state the problem. We develop our analytical model in Section 5. In Section 6, we show and discuss the numerical results as well as illustrate how to use the developed model to configure the network optimally. Conclusion is given in Section 7.

## 2. Brief Introduction to LoRaWAN

### 2.1. Architecture

Let us briefly introduce the main aspects of LoRaWAN operation, while the detailed description can be found in [4].

A typical LoRaWAN [6] network consists of a server, gateways (GWs) and end devices, called motes. Being connected to the server via an IP network, and to motes via LoRa links, the GW acts as a relay between the motes and the server.

LoRaWAN standard describes three classes of motes.

Class A are the simplest motes that support only the basic LoRaWAN functionality. Nevertheless, they support bi-directional acknowledged communications with the GW. For uplink transmission, the motes access the channel in a way similar to unslotted ALOHA. The GW can transmit in the downlink only after successful uplink transmissions. For a low network load, class A provides the lowest energy-consumption for the motes, that is why class A motes are widely used in practice and considered in the paper.

Class B implements bi-directional communication with scheduled downlink receive slots. The GW disseminates schedule information in beacons.

Class C motes listen to the channel continuously, thus providing the lowest downlink latency, but require extremely high power consumption.

### 2.2. PHY

LoRaWAN uses a PHY layer protocol named LoRa which is based on a variant of Chirp Spread Spectrum modulation [7].

Each LoRa symbol can represent $2^{SF}$ values, where SF is the spreading factor. The duration of the symbol $T_{s}$ depends on SF and bandwidth BW as $T_{s}=\frac{2^{SF}}{BW}$. The set of available data rates depends on regional specifications. Table 1 lists possible modulation and coding schemes (MCSs) [8] for European EU 863–880 MHz ISM (industrial, scientific, and medical) band.

The used MCSs have two important features. First, a LoRa mote can correctly receive two overlapping transmissions with different spreading factors in the same channel. Second, the primary LoRa vendor claims that even in case of two simultaneous transmissions with the same spreading factor, a mote can correctly receive the most powerful one, provided that the difference in signal power is higher than 3 dB [9].

### 2.3. Frame Formats

Since LoRaWAN is designed for low-power low-rate short-packet communications, heavy TCP/IP protocols are hardly applicable. Thus, LoRaWAN proposes a lightweight stack which can be directly used by sensor and actuator applications to communicate with the network server.

**Figure 1:**
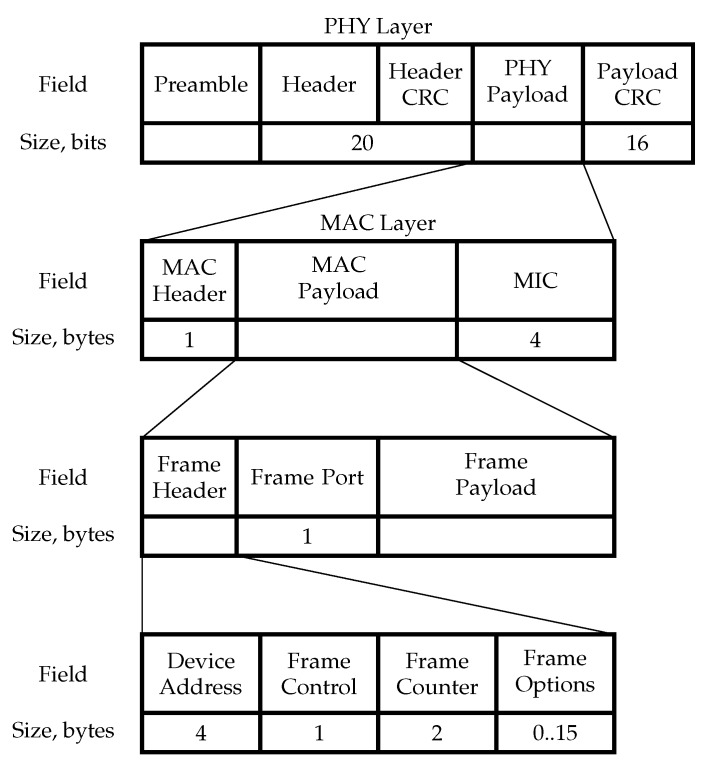
LoRaWAN Frame Format [4].

At the PHY layer, a LoRaWAN frame (see Figure 1) starts with a preamble with a typical duration of 12.25 $T_{s}$. The main goal of the preamble is synchronization. Also, the preamble determines the packet SF, since it is exactly the same as of the preamble.

The total length of the PHY Header and Header CRC is 20 bits. They are encoded with the most reliable code rate of $\frac{1}{2}$. The PHY header carries information about the payload length and whether the Payload 16-bit CRC is present in the frame. Note that the Payload CRC is only present in uplink frames.

At the MAC layer, the Header includes a protocol version and message type, e.g., whether it is a data or a management frame, whether it shall be acknowledged, etc. message integrity check (MIC) is used as a digital signature of the message.

In the MAC payload, the frame header includes the following information.

- Device address, the first 8 bits of which identify the network, while the other 24 bits are assigned dynamically while joining the network.
- Frame Control information, such as whether to use the data rate specified by the GW for uplink transmission, whether this message acknowledges the reception of the previous message, whether the GW has more data for the addressee.
- Sequence number.
- Frame options, which can contain commands used to change data rate, transmission power, validate the connection, etc.

Frame port serves a similar purpose to that of TCP or UDP protocols, i.e., it allows distinguishing several flows generated by various applications. If it equals zero, the frame payload contains MAC commands instead of user data.

### 2.4. Channel Access

**Figure 2:**
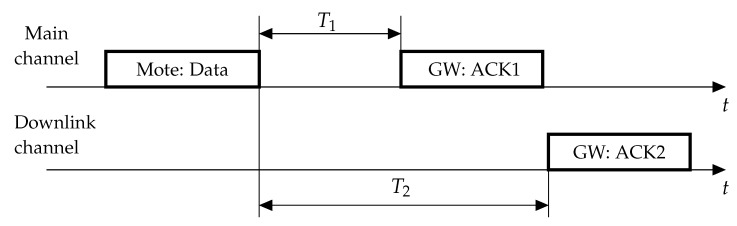
Data transmission and acknowledgment in a LoRaWAN network.

A LoRaWAN network simultaneously operates on several wireless channels. For example, in Europe LoRaWAN devices by default use three main channels and one downlink channel. To transmit a data frame, each mote randomly selects one of the main channels (see Figure 2). The MCS used for the transmission of data frames is determined for each mote by the network server. The LoRaWAN standard mentions the adaptive data rate (ADR), which is the MCS and transmission power allocation to the motes in such a way that the devices use the fastest MCS possible. However, the standard does not define any exact algorithm for MCS selection to implement the ADR, and neither does it present any algorithms to configure the MCSs to provide any specific QoS guarantees in the network, leaving just a framework for implementation-dependent solutions.

Having received the frame, the GW transmits two acknowledgments (ACKs). The first ACK is sent $T_{1}$ after the frame reception in the same channel where data was transmitted. By default, the MCS of the ACK is the same as for data. However, the specification allows reducing it by a configurable offset. The second ACK is sent typically at the lowest possible data rate after timeout $T_{2}=T_{1}+1\;s$ in the downlink channel.

If the mote does not receive any ACK, it makes a retransmission. The standard recommends [8] making a retransmission in a random time drawn from interval [1,1+W] seconds, where W=2.

## 3. Prior Art on LoRaWAN Analyses and MCS Allocation

The peculiarities of the LoRa MCSs make their impact on the overall LoRaWAN performance twofold. First, the lower the MCS, the more reliable is the transmission to/from the edge motes. Second, since all the MCSs, except for MCS6, have different spreading factors, by allocating different MCSs to various motes, the network server can orthogonalize their transmissions and reduce the collision probability. Thus, the main question is how exactly the performance of a LoRaWAN network depends on the traffic and MCS allocation policy. Note that the MCS allocation policy significantly affects network performance.

Although the LoRaWAN specification was released in 2015, LoRa/LoRaWAN, including the issue mentioned above, has already been extensively studied, see [10,11,12,13,14,15,16,17,18,19,20,21].

Paper [10] presents an analytical model of LoRaWAN class A that allows finding the latency, collision probability, and the service ratio for a LoRaWAN network. The model calculates the latency by considering the waiting process as an M/D/1 and an M/D/c queue, while the collision probability is found in the same way as for a multichannel ALOHA random access scheme. The developed model describes only the uplink unacknowledged traffic and does not take into account downlink traffic, retransmissions, and capture effect.

Paper [12] is explicitly dedicated to the power, channel, and spreading factor allocation problem. The authors use a simple ALOHA-like model of the LoRaWAN network to develop an algorithm that solves the problem in the following way. Firstly, the motes are sorted by their path loss and split into equal groups, the number of which equals the number of available channels. Each group is assigned its separate channel, and within the groups, the percentage of motes using SF *s* is proportional to $\frac{s}{2^{s}}$, which corresponds to the solution of the optimization problem to minimize the maximal collision probability among all the SFs. The algorithm also checks the feasibility and updates the decision if a mote is assigned to a too-low SF with given power limitations.

Paper [19] extends the research from [12]. Firstly, the allocation problem considers the possibility of motes to use different bandwidths and code rates, so the percentage of motes using SF *s*, bandwidth *b* and code rate *c* is proportional to $\frac{s\times b\times c}{2^{s}}$. Secondly, the authors have proposed a region concept to allocate SFs within a cell, where nodes are assigned to a region based on their RSSI and within their regions, they are allocated SFs according to the given ratios. Thirdly, the fairness of resource allocation is considered, and the fairness of resource allocation is achieved by the developed power allocation algorithm, which tries to equalize the RSSI of the motes, assigning low transmit power to the motes with a strong signal and high transmit power to the motes with a weak signal. Experimental results in comparison with [12] generally show higher Jain’s fairness index of the data extraction rate, its stability with an increasing number of nodes in the network and lower energy consumption, which is caused by the fact that the power allocation algorithm equalizes the power of motes to the lowest one. At the same time, the average data extraction rate is lower than in [12].

Similar problems are studied in [13], where the authors propose two algorithms for SF allocation in LoRaWAN networks. The first algorithm, EXPLoRa-SF, assigns the lowest available SF to nodes having the best RSSI, and the number of nodes assigned to each SF is the same for all SFs. The second algorithm, EXPLoRa-AT, equalizes the time-on-air between the different SFs by keeping the number of nodes assigned to each SF inversely proportional to its time-on-air. The performance of these two algorithms has been compared to the performance of the ADR algorithm, and the results show that EXPLoRa-SF outperforms ADR, while EXPLoRa-AT provides even better performance in case of the highly loaded system.

The strategy to split the motes into the groups and keep the number of motes assigned to each SF inversely proportional to their time-on-air is also validated in [11], where the authors consider the impact of possible non-orthogonality of LoRa signals with different spreading factors. Simulation and USRP experiments show that, although the signals with different SFs are orthogonal in idealistic scenarios, the discretization and implementation peculiarities may make these signals non-orthogonal depending on the spreading factors and power levels of the transmitted and interfering signals. For example, a signal transmitted at SF=12 cannot be decoded successfully if it is interfered by a signal with SF=7…11 and the SINR is less than −16 dB. As a result, simultaneous transmission of frames at different SFs may result in frame collisions if the difference in their powers is very high. The authors also propose and show the benefits of balancing the time-on-air between different SFs by doubling the number of nodes using an SF when switching to the next lower SF.

The influence of the imperfect orthogonality of SFs in LoRa is further studied in [16]. Here the authors use the results from [11] to calculate the probability of successful frame transmission and the coverage probability in a LoRa network, in which devices use various SFs and frames transmitted at different SFs can collide if their powers differ significantly. The obtained results show that in a large area LoRaWAN network with a radius of tens of kilometers, the impact of inter-SF interference can be rather high. At the same time, at a low distance, less than 1 km, the influence of inter-SF interference is less notable.

Another research [17] studies the impact of the inter-SF interference on the throughput of the network. It shows that the throughput calculated considering the inter-SF interference is approximately 10% lower than the throughput obtained only considering the same-SF interference. However, such a difference can be obtained only for high network load, while for a network utilized by less than 50% the difference becomes negligible.

Paper [18] targets on maximizing the minimal rate among the motes which transmit their data to a GW by allocating appropriate MCSs and power levels. The rate is calculated according to Shannon’s formula, taking into account the Rayleigh fading model. Maximization of the minimal rate by channel and MCS assignment is not a convex optimization problem, so we proposed to solve it in two steps: firstly to assign the channels to motes and then to allocate the power to motes which transmit within the same channel. The first step is accomplished using a matching theory-based algorithm, and the second step is solved using a bisection search approach after noticing that the power allocation problem is quasi-concave. The main drawback of the paper is that the authors consider the network from the PHY perspective only and disregard all the MAC layer peculiarities. They calculate the rate assuming that all the users transmit simultaneously, and such essential aspects as the transmission durations, their variability at different spreading factors and the collision probability are not taken into account at all.

Paper [20] considers the SF allocation problem in a LoRaWAN network. The authors develop a mathematical model of LoRa transmission in a scenario, where motes are uniformly spread within a circle around the GW. They consider that the total flow of packets in the network is Poisson and find the collision probability using the approach for modeling ALOHA networks. They use a log-distance path-loss model to calculate the arriving signal powers from the nodes and thus find the probability that a frame is successful even if there is a collision. It should be noted that both inter-SF and intra-SF collisions have been considered, i.e., they assumed that a frame could be lost due to a frame with a different spreading factor if the power of the interfering signal is too high. They find the average success probability among all motes and state an optimization task to maximize the number of motes in the network such that the average success probability is above the requested level while varying the percentage of nodes that use specific spreading factors. The results show that optimal SF allocation can increase the potential number of served users by up to 705% comparing with the uniform SF allocation. It should be noted, though, that the optimization results have shown that the considered capacity metric is maximized when all the users in the network are assigned the two lowest SF values, and the rest of the SFs are not used at all, which seems counter-intuitive and perhaps needs additional investigation.

In [22], the authors propose to improve the performance of LoRa networks by message replication and using GWs with multiple receive antennas, thus achieving diversity in time and space.

In [21], the authors highlight the critical limitation of LoRaWAN networks, related to the GW having a limited number of demodulation paths. The authors develop a mathematical model that takes into account this limitation and derive the frame success probability, coverage probability, and the throughput. They also investigate three SF allocation algorithms: the uniform allocation scheme, distance-based allocation scheme, and the equivalent load scheme. The equivalent load scheme is similar as the one studied in [11,12] and provides the highest throughput.

Summing up, the problem of MCS allocation for improving overall throughput has been widely studied in the literature. For such a target as cumulative throughput, a clear and easy-to-implement solution exists: the number of motes transmitting with an SF shall be inversely proportional to the time-on-air for such MCS. Since for the frames of a fixed length, time-on-air almost doubles every time we increase SF by one (reduce the index of MCS by one), the number of motes shall be distributed proportionally to $2^{-<\mathrm{Index}\mathrm{of}\mathrm{MCS}>-1}$. However, for other targets, the problem becomes more complicated.

Specifically, so far in the literature, the problem of the optimal satisfaction of different QoS for various motes has not been considered yet.

## 4. Problem Statement

We study a network of LoRaWAN class A devices, which has a GW and *G* groups of motes, group *g* having $N_{g}$ motes. The motes transmit frames to the GW, and the frames arrive at the motes according to a Poisson process, which for the motes of a group *g* has a parameter $\lambda_{g}$, further referred to as the network load. The GW acknowledges all received data frames with ACKs carrying no payload. When a mote receives no ACK for the transmitted frame, it makes retransmission in a random time from 1 to 1+W seconds, and every frame can be transmitted up to RL times. The GW discards a frame if it cannot deliver it after RL transmission attempts. We also consider that if a mote generates frames while transmitting another frame, then after the end of the second receive window it starts processing the most recently generated frame, and the old frames are discarded. Such an assumption is related to the simplicity of the motes, i.e., their buffers are small.

Similarly to [5], we take into account the capture effect, i.e., we consider that a frame can be received correctly even if it intersects with other frames, but its power surpasses the total power of noise and interfering signals with the same MCS by a co-channel rejection parameter CR dB. Additionally, in contrast to [5], we take into account transmission failures which are caused by some random noise in the unlicensed band rather than by collisions with other LoRaWAN transmissions. For that, we introduce the probability *q* that a frame transmitted without collisions cannot be received.

The devices use *F* main channels and a downlink channel. For the transmission in main channels, the GW assigns MCSs from 0 to *M* to the motes, while in the downlink channel the ACKs are sent on MCS 0. The motes are spread uniformly within a circle with radius *R* around the GW, and the circle radius is low enough to allow all the motes to use any of the available MCSs.

The frames generated by a mote of a group *g* need to be delivered with PLR lower than $PLR_{g}^{QoS}$. Since transmissions at different MCSs almost do not collide [17], and PLR depends on the load at the corresponding MCS, we can satisfy QoS by appropriate MCS allocation, if such allocation exists. Without loss of generality, in the paper, we consider a case when the motes are close enough to the GW, so each of them can use any of the MCSs.

To find such an MCS allocation that satisfies requirements on $PLR_{g}^{QoS}$, we develop a mathematical model that finds PLR as functions of the traffic generated by the motes transmitting with a given MCS. Also, the model allows finding the distribution of the data delivery time, i.e., the time between the instance when data is generated until the instance when the ACK is received.

## 5. Mathematical Model

### 5.1. General Description

We design a mathematical model of data transmission in a LoRaWAN network by extending our ideas published in [5].

Similarly to [5], we distinguish between the initial transmission attempts and the retransmissions and find the probabilities of successful transmission attempts for them separately. However, we extend the model from [5], taking into account possible transmission failures caused by random noise rather than by collisions only. Such extension slightly modifies formulas for the initial transmission attempt derived in Section 5.2. At the same time, the model extension significantly changes the formulas describing retransmissions, see Section 5.3. The probabilities of successful initial transmission and retransmission are found under the assumption that the rate of the transmission attempts is low enough to avoid the avalanche effect described in [4]. The accuracy bound of the model is studied in detail in Section 5.4. A corollary of this assumption is that we can calculate the probability of successful ACK transmission in a way different from [5]. This way allows analyzing data transmission at each MCS separately, ignoring that the flow of the second ACKs depends on successful transmissions at other MCSs and in other channels.

Another assumption made is that signals transmitted at different MCSs are orthogonal to each other. This assumption results from the small radius *R* of the network, since, as shown in [16], the impact of inter-SF interference is very low at a small distance between devices. At the same time, as shown in [17], the impact of inter-SF interference becomes considerable only at the high network load, but at the high network load, the above-mentioned avalanche effect becomes much more important from the collision point of view.

Whether both colliding frames are damaged depends on their signal strength. To compute the probability that a frame can survive in a collision, we need to know the locations of the motes around the GW. In contrast to [5], we consider a case when the motes are close enough to the GW and address this issue in Section 5.5.

In Section 5.6, given probabilities of successful initial transmission and retransmissions, we find PER, similarly to [5]. Finally, we design original methods to find PLR and delay distribution in Section 5.7 and Section 5.8, respectively.

### 5.2. Initial Transmission Attempt

Let us consider a mote that uses MCS *i* for transmission. Its initial transmission of a data frame is successful if the GW receives the frame and the mote receives at least one of the ACKs from the GW. The initial transmission attempt at MCS *i* is successful with probability

(1) $$P_{i}^{S,1}=P_{i}^{Data}P_{i}^{Ack},$$

where $P_{i}^{Data}$ is the probability that the GW receives the data frame, and $P_{i}^{Ack}$ is the conditional probability that the mote receives at least one ACK provided that the GW receives the data frame.

Let $T_{i}^{Data}$ and $T_{i}^{Ack}$ be the durations of the data frame and the ACK at MCS *i*, respectively.

Since the transmissions at different MCSs are orthogonal, we can consider transmissions in different main channels and at different MCSs independently. Let us denote the distribution of MCSs among the motes as $p_{i}$ and the traffic intensity which corresponds to one main channel and this MCS as $r_{i}=\frac{\lambda p_{i}}{F}$, where $\lambda =\sum \lambda_{g}$ is the total network load.

We find $P_{i}^{Data}$ as follows.

Let data frame transmission start at time 0. The transmission does not suffer from collisions in two cases.

No collision. The first one happens when the data frame collides with neither a frame sent by another mote nor the ACK sent by the GW. If no collision occurs, the transmission is not damaged by noise with probability 1−q.

Collisions with data frames do not occur if no frame starts in the interval $\left[-T_{i}^{Data},T_{i}^{Data}\right]$. The probability of this event is $e^{-2r_{i}T_{i}^{Data}}$ since the flow of the new frames is Poisson with intensity $r_{i}$.

Collisions with ACKs do not happen if no ACK starts in the interval $\left[-T_{i}^{Ack},0\right]$, since the GW cancels a pending ACK transmission if the GW is receiving a data frame at the same MCS, at the same channel, and at the same time instant when the GW needs to start the ACK. The probability of such an event is $e^{-r_{i}P_{i}^{Data}T_{i}^{Ack}}$ since the flow of ACKs is also Poisson but has an intensity $iP_{i}^{Data}r_{i}$ because ACKs are transmitted only for successful data frames.

Collisions. The second case happens when the data frame intersects with frames from some other motes but has a power high enough to be decoded by the GW. In this case, if the frame collides with *k* other frames, the transmission is successful with some probability $V_{i,k}^{GW}$ which is derived in Section 5.5.

Since the new frames are generated according to the Poisson flow, the probability of *k* motes generating their frames in the interval $\left[-T_{i}^{Data},T_{i}^{Data}\right]$ equals $e^{-2r_{i}T_{i}^{Data}}{\left(2r_{i}T_{i}^{Data}\right)}^{k}/k!$.

Summing up, we obtain

(2) $$P_{i}^{Data}=\left(1-q\right)e^{-\left(2T_{i}^{Data}+P_{i}^{Data}T_{i}^{Ack}\right)r_{i}}+\sum_{k=1}^{N-1}\frac{{\left(2r_{i}T_{i}^{Data}\right)}^{k}}{k!}e^{-2r_{i}T_{i}^{Data}}V_{i,k}^{GW},$$

where $N=\sum N_{g}$ is the total number of motes.

When the GW receives a data frame, it sends two ACKs. The probability that the mote receives at least one sent ACK is calculated as follows:

(3) $$P_{i}^{Ack}=P_{i}^{Ack1}+P_{i}^{Ack2}-P_{i}^{Ack1}P_{i}^{Ack2},$$

where $P_{i}^{Ack1}$ and $P_{i}^{Ack2}$ are the probabilities of the first and the second ACKs being successful if the corresponding data frame is sent at MCS *i*.

The probability $P_{i}^{Ack1}$ is found similarly to $P_{i}^{Data}$.

We denote the ACK transmission start time as 0 and consider two cases.

1. No collisions The ACK does not collide with any data frames if no data frames are generated within $T_{i}^{Ack}$ after the ACK starts.
  Apart from that, to avoid collisions, no motes shall start data transmission $T_{i}^{Data}$ before the ACK. However, if $T_{1}<T_{i}^{Data}$, the data transmission starts during the interval $\left[-T_{i}^{Data},-T_{1}\right]$ and corrupts the data frame acknowledged by the considered ACK. It means that the ACK shall not be sent at all and we need to exclude the interval $\left[-T_{i}^{Data},-T_{1}\right]$.
  Thus, we obtain that in this case, the other motes shall not generate data frames within the interval $\left[-\min\left(T_{1},T_{i}^{Data}\right),T_{i}^{Ack}\right]$.
2. The mote can also successfully receive the ACK if some data frames are generated in the interval $\left[0,T_{i}^{Ack}\right]$, but the ACK has sufficient power to overcome the noise and the interference.

Let $V_{i,k}^{Mote}$ be the probability of total interfering signal from *k* motes and noise (in dBm) having less power than the ACK plus co-channel rejection CR. Unlike [5], we also consider the probability that without collisions a mote correctly receives the signal with probability 1−q.

Summing up, we obtain

(4) $$P_{i}^{Ack1}=\left(1-q\right)e^{-(\min\left(T_{1},T_{i}^{Data}\right)+T_{i}^{Ack})r_{i}}+\sum_{k=1}^{N-1}\frac{{\left(r_{i}T_{i}^{Ack}\right)}^{k}}{k!}e^{-r_{i}T_{i}^{Ack}}V_{i,k}^{Mote},$$

Since the GW transmits the second ACK in a separate channel, the ACK cannot collide with any data frames. However, the GW can discard the ACK because of the transmission of an ACK to another data frame delivered at any other MCS or on any other channel. Thus, to be successful, the ACK shall not start in the interval $\left[-T_{0}^{Ack},0\right]$ relative to the beginning of another ACK. In this paper, when calculating $P_{i}^{Ack2}$, we used the assumption made in Section 5.1, and natural assumption that *q* is small. These assumptions allow us to consider that the probability of another data frame delivery being successful is close to 1, in contrast to [5]. Although such an assumption slightly limits the area of applicability of the model, it allows us to calculate $P_{i}^{Ack2}$ for MCS *i* independently from the distribution of the motes per MCSs:

(5) $$P_{i}^{Ack2}=\left(1-q\right)e^{-T_{0}^{Ack}\left(\lambda -r_{i}\right)},$$

where we also consider the probability 1−q of the ACK not being damaged by the noise.

### 5.3. Retransmissions

Let a mote transmit a data frame but receive no ACK. This case may be caused by the unsuccessful data frame or ACK transmissions, or by an ACK discarded because of another incoming transmission. In this case, the mote retransmits the data frame. For that, the mote randomly selects a channel and waits for a random time from 1 to 1+W seconds. Then it sends the data frame again.

In our analysis, we consider only the retransmissions that are due to the noise or a collision of two frames. In other words, we neglect the collisions of more than two frames. Thus we can find the probability of successful retransmission, but at the same time does not induce a significant error in our estimation of PLR, PER, and the delay distribution, as shown in Section 6. Unlike [5], we take into account that even without collisions, the retransmission can be unsuccessful because of the random noise in the channel, which complicates the formulae.

We study four cases why retransmission may occur: (i) no collision, i.e., the retransmission is caused just by noise, (ii) collision: one of the frames is received because of stronger power, (iii) collision: although the stronger frame could be received but for the noise, it is lost, (iv) collision: both frames have insufficient power to be received.

In the first case, the retransmission is caused by random noise. Let ζ be the probability that either data frame or both ACKs are damaged by noise. It is a complement to the case when the data frame and at least one ACK are not damaged by noise. The GW receives the data frame with a probability of 1−q. The mote receives at least one ACK with the probability $2\left(1-q\right)-{\left(1-q\right)}^{2}$ since the transmissions of the two ACKs are independent. Thus, we obtain

(6) $$\zeta =1-\left(1-q\right)\left[2\left(1-q\right)-{\left(1-q\right)}^{2}\right].$$

Collisions and noise are independent. Since the first transmission attempt is successful if both the noise and collisions do not occur, the probability $P_{i,1}^{S}$ of successful transmission is the product of the probability of the absence of collisions and the probability of the absence of the noise (1−ζ). Thus, we can find the probability of the absence of collisions as $\frac{P_{i,1}^{S}}{1-\zeta }$, and probability that the retransmission is due to noise only equals $\zeta \frac{P_{i,1}^{S}}{1-\zeta }$.

The second case corresponds to a collision when one frame is received since its power $u_{A}$ is much higher than the power $u_{B}$ of another frame: $u_{A}>u_{B}+CR$, and the random noise does not damage the first frame. As a result of such a collision, the first mote successfully delivers its frame, and the second one makes retransmission.

Let $V_{i}^{one}$ be the probability of such a power relation between two motes. Then the probability of such a case is the product of the probabilities of the following events: (i) a collision occur with the probability $\left(1-\frac{P_{i,1}^{S}}{1-\zeta }\right)$, (ii) $u_{A}>u_{B}+CR$, which happens with probability $V_{i}^{one}$ determined in Section 5.5, (iii) no noise damages the transmission of the first mote with probability $\left(1-\zeta \right)$. Summing up, we obtain that the probability of such a case equals

(7) $$\left(1-\frac{P_{i,1}^{S}}{1-\zeta }\right)V_{i}^{one}\left(1-\zeta \right).$$

The third case is similar to the second one, with the only difference that the first frame is damaged by noise. It means that in the last multiplier in (7) we need to replace $\left(1-\zeta \right)$ with ζ. Thus we obtain that the probability of the third case is $\left(1-\frac{P_{i,1}^{S}}{1-\zeta }\right)V_{i}^{one}\zeta$.

The fourth case stands for a situation, when the difference in signal strength of both frames is less than CR: $|u_{A}-u_{B}|<CR$. So none of the frames can survive in a collision. Such a relation between signal strengths happens with probability $V_{i}^{both}$ determined in Section 5.5. So the probability of the fourth case is $\left(1-\frac{P_{i,1}^{S}}{1-\zeta }\right)V_{i}^{both}$.

In the last two cases, both frames are transmitted again, and there is a high probability of a repeated collision since the retransmissions are made within a rather short delay, comparing with the data frame duration (W=2 s vs. $T_{0}^{Data}\approx 2.4\;s$). Let us find this probability similarly to [5].

**Figure 3:**
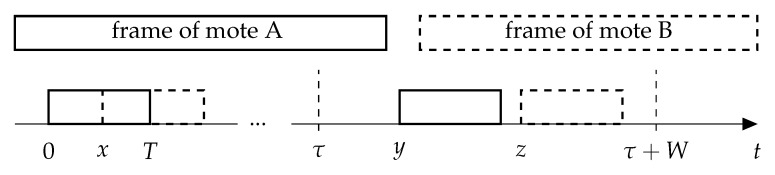
Retransmission.

Let the frames sent by motes A and B begin at time 0 and *x*, respectively (see Figure 3). The collision does not occur if the motes retransmit on different channels. Otherwise, with probability $\frac{1}{F}$, the motes choose the same channel. In this case, let the retransmitted frames by motes A and B begin at times *y* and *z*, respectively. The distribution of *y* is uniform from τ to τ+W, where τ is the duration of the frame and the ACK timeout. The distribution of *z* is also uniform from τ+x to τ+x+W. A new collision happens during the retransmission if the frames intersect with each other or with the ACK. Let us define the indicator of such an event $f\left(y,z,T_{i}^{Data},T_{i}^{Ack}\right)$:

(8) $$\begin{array}{cc} f\left(y,z,T_{i}^{Data},T_{i}^{Ack}\right) & =𝟙\left(y<z\leq y+T_{i}^{Data}\right)+𝟙\left(y+T_{i}^{Data}+T_{1}<z\leq y+T_{i}^{Data}+T_{1}+T_{i}^{Ack}\right)+\\ & +𝟙\left(z\leq y\leq z+T_{i}^{Data}\right)+𝟙\left(z+T_{i}^{Data}+T_{1}\leq y\leq z+T_{i}^{Data}+T_{1}+T_{i}^{Ack}\right), \end{array}$$

where 𝟙(X) is one, if the event *X* occurs, and 0, otherwise. Notably, four events of (8) do not overlap.

We use it to find the probability of a new collision:

(9) $$P_{i}^{c}=\frac{1}{F}\;\cdot \;\frac{\int_{-T}^{T}r_{i}e^{-r_{i}x}\int_{0}^{W}\int_{x}^{W+x}\frac{f\left(y,z,T_{i}^{Data},T_{i}^{Ack}\right)}{W^{2}}dzdydx}{\int_{-T}^{T}r_{i}e^{-r_{i}x}dx}.$$

In the numerator, the outer integral includes all the possible intervals between frames by motes A and B that lead to collision, while the inner integrals contain all the possible retransmission times for the motes. In the denominator, we have the collision probability which is needed to obtain the conditional probability of a new collision, given that the previous collision happened.

Taking into account all the cases above, we obtain the resulting probability $P_{i,Re}^{Data}$ of successful delivery of data frame during a retransmission attempt as the fraction of the probability that retransmission occurs and the data frame is delivered, and the probability that retransmission occurs.

A retransmission occurs with the total probability of four cases:

(10) $$\zeta \frac{P_{i,1}^{S}}{1-\zeta }+\left(1-\frac{P_{i,1}^{S}}{1-\zeta }\right)\times \left(V_{i}^{one}\left(1-\zeta \right)+V_{i}^{one}\zeta +V_{i}^{both}\right).$$

In the first two cases, the probability of the delivery of the data frame is the same as for the first transmission attempt, namely $P_{i}^{Data}$. In the third and fourth cases it equals $\left(1-P_{i}^{c}\right)P_{i}^{Data}$ since the collision between the two colliding frames may repeat. If it does not repeat, the frames can be damaged by other transmissions or the random noise, similarly to the first transmission attempt.

Thus, we obtain:

(11) $$P_{i,Re}^{Data}=\frac{P_{i,1}^{S}\frac{\zeta }{1-\zeta }+\left(1-\frac{P_{i,1}^{S}}{1-\zeta }\right)\times \left[V_{i}^{one}\left(1-\zeta \right)+\left(V_{i}^{one}\zeta +V_{i}^{both}\right)\times \left(1-P_{i}^{c}\right)\right]}{P_{i,1}^{S}\frac{\zeta }{1-\zeta }+\left(1-\frac{P_{i,1}^{S}}{1-\zeta }\right)\times \left(V_{i}^{one}+V_{i}^{both}\right)}P_{i}^{Data}.$$

The probability of successful retransmission $P_{i}^{S,Re}$ is obtained according to (1), where $P_{i}^{Data}$ is replaced with $P_{i,Re}^{Data}$.

### 5.4. Accuracy Bound of the Model

The developed model uses the assumption that the traffic in the network is not very high. Specifically, the rate of the transmission attempts is low enough to avoid the avalanche effect described in [4]. The avalanche effect is a situation when motes that make a retransmission are more likely to be involved in a new collision and, moreover, as long as they try to resolve the collisions, the probability of unsuccessful transmission increases for another mote making the first transmission attempts, so as a result most motes in the network are involved in the collision resolving process. Such a situation becomes possible for such a network load when new frames come so often that every retransmission results in a collision with a newly generated frame. To obtain it, we divide *F* by the average retransmission duration:

(12) $$\lambda^{*}=F{\left(\sum_{i=0}^{R}p_{i}\left(T_{i}^{Data}+T_{2}+T_{0}^{Ack}+1+\frac{W}{2}\right)\right)}^{-1}.$$

As this load corresponds to the situation when our model assumption does not hold, we further use it as an accuracy bound for the model.

### 5.5. Specific Case

The formulae in Section 5.2 and Section 5.3 are written in a very general way and depend on the values $V_{*}^{*}$. To define them, we have to consider a specific scenario, i.e., the deployment of motes around the GW and the channel model.

For simplicity, we consider a channel with a log-distance path-loss and with a constant probability *q* of the signal to be damaged by random noise. For such a case, if the power of the transmitted signal equals $w_{tx}$ dBm, the power of the received signal equals

(13) $$w_{rx}\left(d\right)=C_{1}-C_{2}\mathrm{lg}\left(d\right),$$

where lg(x) is base 10 logarithm of *x* and *d* is the distance between the transmitter and the receiver. We consider the well-studied Okumura-Hata model [23] since it accurately describes the propagation of signals for the range of 100–1500 MHz in exterior environments, and it is not much different from the empiric channel models developed especially for LoRa links, e.g., [24]. For the Okumura–Hata model, the constants are defined as follows

(14) $$C_{1}=w_{tx}-69.55-26.16\mathrm{lg}\left(f\right)+13.82\mathrm{lg}\left(h_{GW}\right)+3.2{\left(\mathrm{lg}\left(11.75h_{Mote}\right)\right)}^{2}-4.97,$$

and

(15) $$C_{2}=44.9-6.55\mathrm{lg}\left(h_{GW}\right),$$

where, $h_{GW}$ and $h_{Mote}$ are the heights of the GW and mote’s antennas, respectively.

Let the motes be spread uniformly within a circle with radius *R*, and the GW be located in the center of this circle. In this case, the pdf of the distance between the mote and the GW equals $\rho \left(r\right)=\frac{2r}{R}$.

**Figure 4:**
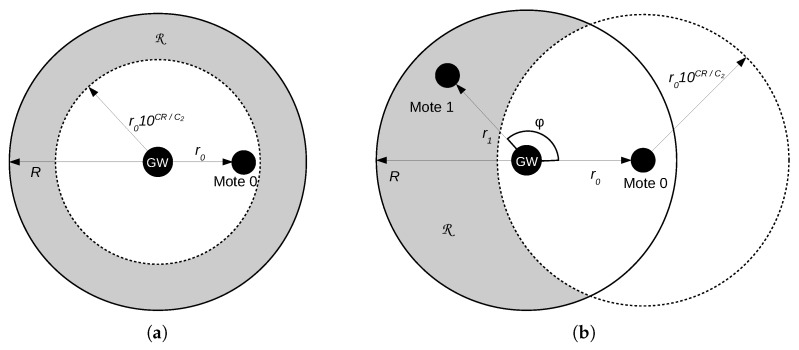
Mote locations for various cases: (**a**) mote 1 location when data frame of Mote 0 is successful in case of collision. (**b**) Mote 1 location when ACK is successful if it collides with data.

Let us consider the intersecting transmission of two motes, mote 0 and mote 1. Transmission of mote 0 is successful if it is not damaged by the random noise and the power of the mote 0 signal exceeds the power of the mote 1 signal at the GW by at least CR dB:

(16) $$\begin{array}{cc} V_{i,1}^{GW}=\left(1-q\right)P\left(w_{1}^{G}<w_{0}^{G}-CR\right) & =\left(1-q\right)P\left(C_{1}-C_{2}\mathrm{lg}\left(r_{1}\right)<C_{1}-C_{2}\mathrm{lg}\left(r_{0}\right)-CR\right)=\\ =\left(1-q\right)P\left(r_{1}>r_{0}10^{\frac{CR}{C_{2}}}\right) & =\left(1-q\right)\int_{0}^{R}\int_{R}\frac{4r_{0}r_{1}}{R^{4}}dr_{1}dr_{0}=\frac{1-q}{2}10^{-2\frac{CR}{C_{2}}}. \end{array}$$

where the outer integral is taken over the possible coordinate $r_{0}$ of mote 0, and the inner integral is taken over such a set of mote 1 coordinates $r_{1}\in R$ that the power condition holds. This set is illustrated in Figure 4a and is defined as

(17) $$R=\left\{r_{1}:0<r_{1}<R\wedge r_{0}\;\cdot \;10^{\frac{CR}{C_{2}}}<r_{1}\right\}.$$

The probability of the motes transmitting with such a power that none of the signals exceeds the interfering one by CR dB equals

(18) $$\begin{array}{cc} V_{i}^{Both}= & P\left(w_{1}^{G}>w_{0}^{G}-CR\wedge w_{0}^{G}>w_{1}^{G}-CR\right)=P\left(w_{0}^{G}-CR<w_{1}^{G}<w_{0}^{G}+CR\right)=\\ = & P\left(C_{1}-C_{2}\mathrm{lg}\left(r_{0}\right)-CR<C_{1}-C_{@}\mathrm{lg}\left(r_{1}\right)<C_{1}-C_{2}\mathrm{lg}\left(r_{1}\right)+CR\right)=\\ = & P\left(r_{0}10^{-\frac{CR}{C_{2}}}<r_{1}<r_{0}10^{\frac{CR}{C_{2}}}\right)=\int_{0}^{R}\int_{R}\frac{4r_{0}r_{1}}{R^{4}}dr_{1}dr_{0}=1-10^{-2\frac{CR}{C_{2}}}. \end{array}$$

In this case, the set R is defined as

(19) $$R=\left\{r_{1}:0<r_{1}<R\wedge r_{0}\;\cdot \;10^{-\frac{CR}{C_{2}}}<r_{1}<r_{0}\;\cdot \;10^{\frac{CR}{C_{2}}}\right\}.$$

Then we find the probability of only one mote’s transmission to be successful:

(20) $$V_{i}^{One}=1-\frac{V_{i,1}^{GW}}{1-q}-V_{i}^{Both}=\frac{10^{-2\frac{CR}{C_{2}}}}{2}.$$

Now we consider an ACK transmission which is interfered by the Mote 1 transmission. The GW’s signal at Mote 0 exceeds the interfering signal by at least CR dB and is received regardless of the noise with the probability:

(21) $$\begin{array}{cc} V_{i,1}^{Mote}=\left(1-q\right)P\left(w_{1}^{M}<w_{0}^{M}-CR\right) & =\left(1-q\right)P\left(C_{1}-C_{2}\mathrm{lg}\left(d_{1}\right)<C_{1}-C_{2}\mathrm{lg}\left(r_{0}\right)-CR\right)=\\ =\left(1-q\right)P\left(d_{1}>r_{0}10^{\frac{CR}{C_{2}}}\right) & =\left(1-q\right)\int_{0}^{R}\underset{R}{\;\int \int \;}\frac{2r_{0}r_{1}}{R^{4}}\frac{d\phi }{\pi }dr_{1}dr_{0}, \end{array}$$

where the outer integral is taken over the possible distance $r_{0}$ from mote 0 to the GW, and the inner integrals are taken over such distance $r_{1}$ and angle ϕ (see Figure 4b) that the power condition holds. This location is defined as

(22) $$R=\left\{r_{1},\phi :0<r_{1}<R\wedge cos\phi \leq \frac{r_{0}^{2}+r_{1}^{2}-r_{0}^{2}10^{\frac{2CR}{C_{2}}}}{2r_{0}r_{1}}\right\}.$$

We also consider that a device cannot receive a frame if the number of interfering devices is more than two, so the probabilities $V_{i,k}^{GW},V_{i}^{One},V_{i,k}^{Mote}$ equal zero for k>1. Such an assumption significantly simplifies the model but does not induce significant errors to the numerical results.

### 5.6. Packet Error Rate

Let us now use the obtained probabilities of successful transmission $P_{i}^{S,1}$ and $P_{i}^{S,Re}$ to find the PER. For that, let us consider an arbitrary frame transmission by a mote which uses MCS *i*. The frame transmission is successful with probability $P_{i}^{S,1}$ if it is an initial frame transmission and with probability $P_{i}^{S,Re}$ otherwise. We find PER by subtracting from 1 the probability of the frame transmission being successful:

(23) $$PER=1-\sum_{i}p_{i}\left(P_{1,i}P_{i}^{S,1}+\left(1-P_{1,i}\right)P_{i}^{S,Re}\right),$$

where $P_{1,i}$ is the probability of an arbitrary frame transmission to be an initial one. To find it, we consider a frame transmission cycle which consists of one initial attempt and several retransmissions. Dividing one—the number of initial attempts in the cycle—by the average number of transmissions in the cycle, we obtain the required probability:

(24) $$P_{1,i}={\left(1+\left(1-P_{i}^{S,1}\right)P_{i}^{G}\sum_{r=0}^{RL-1}{\left(P_{i}^{G}\left(1-P_{i}^{S,Re}\right)\right)}^{r}\right)}^{-1}.$$

### 5.7. Packet Loss Ratio

Let a mote which uses MCS *i* generate a frame and start its transmission. The initial transmission attempt is successful with probability $P_{i}^{S,1}$. Otherwise, with probability $1-P_{i}^{S,1}$ the transmission attempt is unsuccessful and the mote retransmits.

The frame is discarded if another frame is generated during the transmission attempt or during the waiting of the random time for retransmission. If we denote the time of the initial transmission start as 0 and the time of the retransmission start as *x*, then the retransmission happens if the next frame is generated after *x*. Taking into account the uniform distribution of *x* and exponential distribution of the frame arrival time, we find the probability of the frame not being discarded before the retransmission:

(25) $$P_{i}^{G}=\frac{1}{W}\int_{0}^{W}e^{-\frac{\lambda }{N}\left(T_{i}^{Data}+T_{2}+T_{0}^{Ack}+1+x\right)}dx=\frac{N}{W\lambda }e^{-\frac{\lambda }{N}\left(T_{i}^{Data}+T_{2}+T_{0}^{Ack}+1\right)}\left(1-e^{-\frac{\lambda }{N}W}\right),$$

where $T_{i}^{Data}$ and $T_{i}^{Ack}$ are the data and ACK durations at MCS *i*.

If the frame is not discarded until the random waiting time, the mote makes a retransmission which is successful with probability $P_{i}^{S,Re}$. Otherwise, with the probability $1-P_{i}^{S,Re}$ the same situation repeats: the mote waits a random time and either generates a new frame or makes another retransmission. The probability of no new frame being generated before the next retransmission still equals $P_{i}^{G}$ since the new frames arrive according to the Poisson flow. Assuming that the probability of success during retransmission does not change with the number of the retransmission, we find the probability of mote making a successful transmission at the *r*-th retransmission as $\left(1-P_{i}^{S,1}\right){\left[P_{i}^{G}\left(1-P_{i}^{S,Re}\right)\right]}^{r}P_{i}^{G}P_{i}^{S,Re}$.

Finally, we denote the probability of the mote using MCS *i* as $p_{i}$ and find the PLR by substracting from 1 the probability of a successful transmission after any possible number of transmission attempts:

(26) $$PLR=1-\sum_{i=0}^{M}p_{i}\left(P_{i}^{S,1}+\left(1-P_{i}^{S,1}\right)P_{i}^{G}P_{i}^{S,Re}\sum_{r=0}^{RL-1}{\left(P_{i}^{G}\left(1-P_{i}^{S,Re}\right)\right)}^{r}\right).$$

### 5.8. Distribution of the Packet Delivery Time

To find the distribution of the packet delivery time, let us find the delay induced by every transmission attempt, starting with the first one. When a frame arrives at a mote, there are two options. If the mote is not transmitting a frame or waiting for an ACK, it transmits the frame at once. Otherwise, the frame has to wait until the currently processed frame is transmitted. Let $T_{i}^{H}$ be the handshake duration at MCS *i*:

(27) $$T_{i}^{H}=T_{i}^{Data}+T_{2}+T_{0}^{Ack}.$$

So if the last transmission attempt of the previous frame started at time 0 and the new frame arrives at time *t*, the delay is $T_{i}^{H}$ if $t\geq T_{i}^{H}$, i.e., the delay is just the time needed to transmit the frame. If $t<T_{i}^{H}$, then the delay is $T_{i}^{H}+T_{i}^{H}-t$. Considering that the frames arrive according to the Poisson process, we can write the following distribution of delay for the first transmission attempt:

(28) $$F_{0,i}^{d}\left(x\right)=P\left(d<x|first\right)=\left\{\begin{array}{cc} 0, & x<T_{i}^{H},\\ e^{-\frac{\lambda }{N}\left(2T_{i}^{H}-x\right)}, & T_{i}^{H}\leq x<2T_{i}^{H},\\ 1, & x>2T_{i}^{H}. \end{array}\right.$$

With the corresponding pdf:

(29) $$p_{first,i}^{d}\left(x\right)=\delta \left(x-T_{i}^{H}\right)e^{-\frac{\lambda }{N}T_{i}^{H}}+I\left(T_{i}^{H}<x<2T_{i}^{H}\right)\frac{\lambda }{N}e^{-\frac{\lambda }{N}\left(2T_{i}^{H}-x\right)}.$$

Since the retransmissions happen after one second plus a uniformly distributed backoff time, the distribution of the delay induced by a single retransmission equals

(30) $$F_{re,i}^{d}\left(x\right)=P\left(d<x|retransmission\right)=\left\{\begin{array}{cc} 0, & x<1+T_{i}^{H},\\ \frac{x}{W}, & 1+T_{i}^{H}\leq x<1+T_{i}^{H}+W,\\ 1, & x>1+T_{i}^{H}+W, \end{array}\right.$$

and has the following pdf:

(31) $$p_{re,i}^{d}\left(x\right)=I\left(1+T_{i}^{H}\leq x<1+T_{i}^{H}+W\right)\frac{1}{W}.$$

Using these distributions we can find the distribution of time needed to deliver a packet, while making *r* retransmissions:

(32) $$F_{r,i}^{d}\left(x\right)=\int_{0}^{x}p_{re,i}^{d}\left(t\right)F_{r-1,i}^{d}\left(x-t\right)dt,$$

which is a convolution of distribution of time, needed to make previous r−1 retransmissions (0 being the first transmission attempt), and the pdf of delay induced by one more retransmission. The starting point for this recurrent formula is the distribution $F_{0,i}^{d}$ defined in (28).

To find the total delivery time distribution, we sum over all possible MCSs and over all possible numbers of retransmissions, multiplying by the probability of a mote using the considered MCS and the frame being successfully transmitted after *r* retransmissions:

(33) $$P\left(d<x\right)=\sum_{i=0}^{R}p_{i}\left(F_{0,i}^{d}\left(x\right)P_{i}^{S,1}+\left(1-P_{i}^{S,1}\right)P_{i}^{G}P_{i}^{S,Re}\sum_{r=1}^{RL-1}F_{r,i}^{d}\left(x\right){\left(P_{i}^{G}\left(1-P_{i}^{S,Re}\right)\right)}^{r-1}\right).$$

We can use the pdf (29) to find the average delay induced by the first transmission attempt:

(34) $$D_{first,i}=\int_{0}^{\infty }xp_{re,i}^{d}\left(x\right)dx=2T_{i}^{H}-\frac{N}{\lambda }\left(1-e^{-\frac{\lambda }{N}T_{i}^{H}}\right),$$

while for a retransmission, the induced delay is obviously $D_{re,i}=1+\frac{W}{2}+T_{i}^{H}$ since it is a uniform distribution.

The average delivery time after *r* retransmissions is the average delay for one retransmission repeated *r* times. Summing up and averaging over possible MCSs we obtain the total average delay:

(35) $$D=\sum_{i=0}^{R}p_{i}\left(D_{first,i}P_{i}^{S,1}+\left(1-P_{i}^{S,1}\right)P_{i}^{S,Re}P_{i}^{G}\sum_{r=1}^{RL-1}\left(D_{first,i}+rD_{re,i}\right){\left(P_{i}^{G}\left(1-P_{i}^{S,Re}\right)\right)}^{r-1}\right).$$

## 6. Numerical Results

### 6.1. Performance Evaluation

Let us use the developed analytical model to study the dependencies of PER, PLR and the delay on the network load and the co-channel rejection parameter CR. We model a LoRaWAN network consisting of N=1000 sensor motes and one GW. They operate in the 863–880 MHz frequency band and use three main and one downlink channel for transmission. The motes use the MCSs from 0 to R=5, specified in the Table 1. All the transmitted frames have the size of 51 byte, which is the biggest size of a frame that can be transmitted at the slowest MCS in LoRaWAN.

To evaluate network performance, we used the developed mathematical model and a simulation model. The simulation does not have simplifications related to the low rate of the transmission attempts, e.g., the one used to calculate the probability of the second ACK being successful. Thus the simulation does not neglect multiple collisions and can show the avalanche effect. However, it still considers that different MCSs are orthogonal.

**Figure 5:**
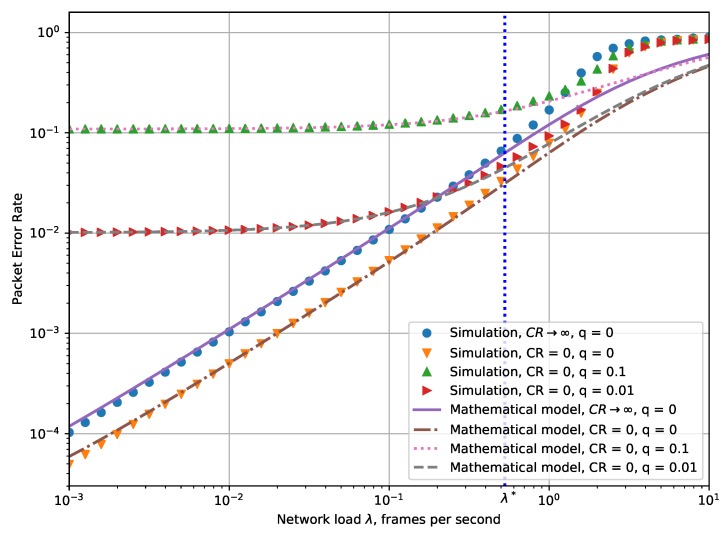
Dependency of packet error rate (PER) on the cumulative network load.

Figure 5 shows the dependency of the PER on the network load λ obtained with the developed mathematical model and simulation.

The plot demonstrates two opposite cases. In the first case, CR→∞, which means that when two frames overlap, none of them can be received. In the second case, CR=0, which means that a frame can be received successfully if its power is higher than the interfering signals. Results for CR=0 are also shown for different probability *n* of the frames to be damaged by the random noise. According to the plot, the developed model can be used to find the PER with sufficient accuracy for any value of CR.

From the plots, one can see that in a case with the capture effect, PER can be up to 50% lower than without the capture effect. At the same time, if we define the maximal network load as the one that provides a given value of PER, then the maximal load can differ by a factor of two when taking into account the capture effect and without it.

According to the numerical results, the level of noise is a significant limiting factor for the LoRaWAN networks, and for low network loads, it becomes the lower bound on PER, i.e., in such scenarios the noise is more important than the interference.

The model provides accurate PER values for the values of network load which are not very high, approximately up to $\lambda^{*}=0.5$ frames per second, while for higher load, the results differ from those obtained with simulation. The error is induced by the assumptions of our mathematical model, specifically, by the fact that the rate of the transmission attempts is low enough to avoid the avalanche effect. However, when the network load is high, the retransmissions are unsuccessful because of the avalanche effect and PER grows up to 100%. Such a high PER means that the motes often retransmit data, which increases the energy consumption of the motes. Therefore, we can conclude that so high network load values are not interesting in practical scenarios.

**Figure 6:**
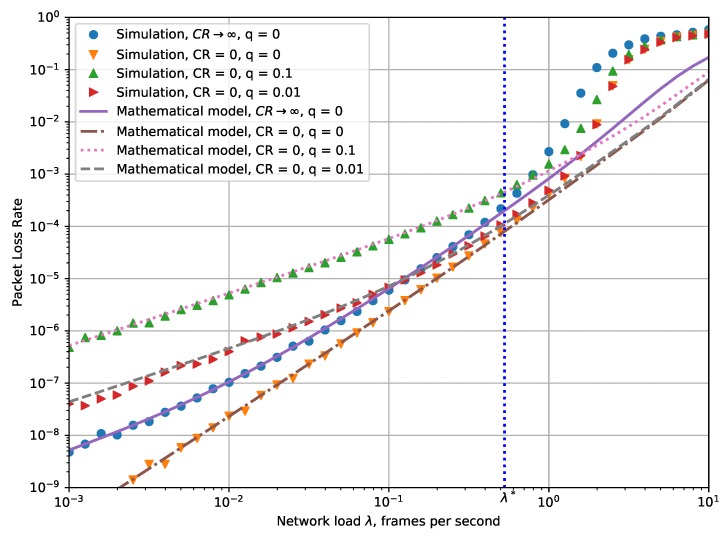
Dependency of packet loss ratio (PLR) on the cumulative network load.

Figure 6 shows the dependency of the packet loss rate (PLR) on the network load. One can see that for the network load less than $\lambda^{*}$, the results obtained with the mathematical model are almost the same as ones provided by simulation. The dependencies of PLR on λ are similar to those of PER, but because of retransmissions, the value of PLR is several orders lower than PER.

Notably, for the probability *q* of the frame to be damaged by random noise greater than 0, the value of PER has a lower bound of *q* and is almost constant for a wide range of λ. For example, for q=0.1, and 0.001<λ<0.5, PER≈0.1,…,0.2. At the same time, the value of PLR changes significantly from $5\times 10^{-7}$ to $5\times 10^{-4}$. The peculiarities of LoRaWAN retransmission rules explain this effect. Because of the small value *W*, the retransmissions can correct failures due to noise, but cannot deal well with collisions.

**Figure 7:**
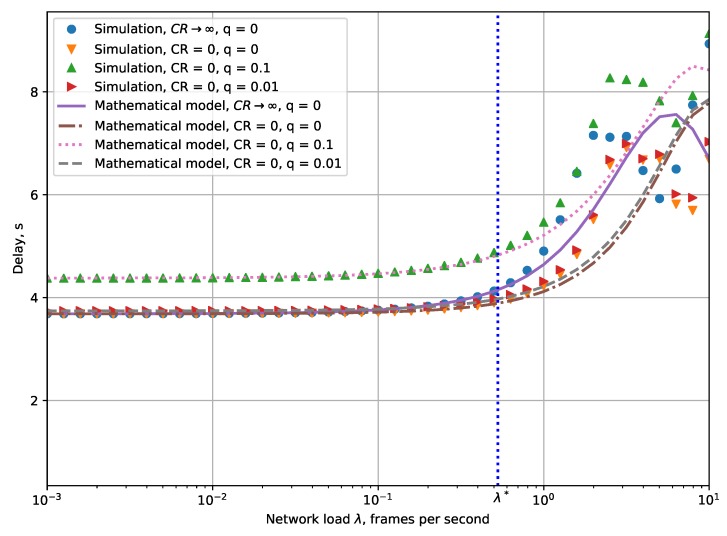
Dependency of the packet delivery time on the network load.

Figure 7 shows the dependency of the average packet delivery time on the network load. When the network load is low, most frames are transmitted at the first attempt and the average packet delivery time equals $\sum_{i=0}^{R}p_{i}D_{first,i}$ in this case. As the network load increases, the percentage of retransmitted frames increases, which results in the growth of the average delivery time. If the network load further increases, the probability of a new frame being generated during the transmission of another frame becomes considerable. In this case, the old frame is discarded, and since the average delivery time is calculated only for the successfully delivered frames, it decreases the average delivery time for very high load (but the corresponding PLR is very high).

**Figure 8:**
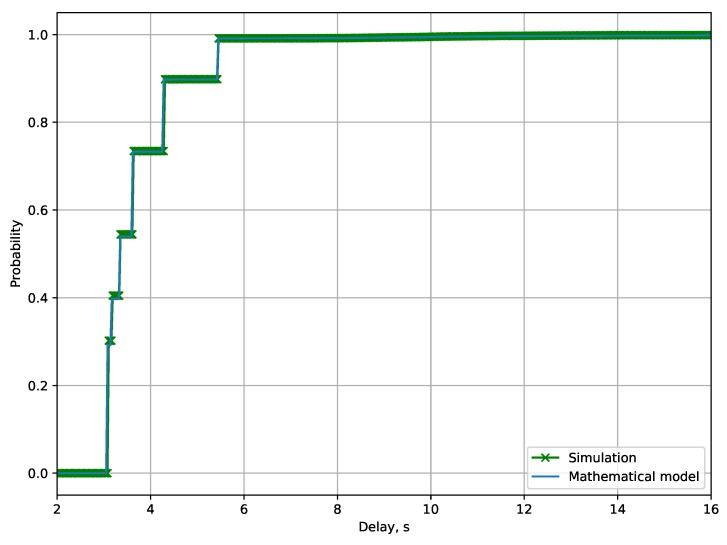
Packet delivery time cumulative distribution function for λ=0.4 frames per second.

Figure 8 shows the cumulative distribution function of the delivery time for network load λ=0.4 frames per second. The CDF for the packet delivery time has a step-like form: each of the six leaps of the CDF corresponds to the situation when a mote transmits a frame successfully at the first try using one of the considered MCSs. With the further increase of the delivery time, the CDF grows gradually, because, in case of an unsuccessful transmission attempt, the mote waits a uniformly distributed backoff. According to the CDF, most of the frames are successfully delivered with the first transmission attempt, and only a small portion of frames are retransmitted. The developed mathematical model can be used to find the packet delivery time distribution with significant accuracy.

### 6.2. MCS Selection

Let us now demonstrate how to use the developed mathematical model for LoRaWAN network planning.

We consider a scenario when the motes are located within a circle with the gateway in the center. The circle radius is 600 m. With such distances, all the motes in all parts of the network can use any MCSs, and the gateway successfully decodes their frames. Let there be three types of motes in the network, each of which has different QoS requirements:

- all motes of the first type generate frames with the cumulative intensity of 0.2 frames per second and require PLR less than $10^{-5}$,
- all motes of the second type generate frames with the intensity of 0.02 frames per second and require PLR less than $10^{-6}$,
- all motes of the third type generate frames with the cumulative intensity of 0.002 frames per second and require PLR less than $10^{-8}$,

If the MCSs are assigned uniformly, (i.e., $p_{i}=1/6$), then if we sum the load we obtain the packet loss rate $PLR\approx 4.1\times 10^{-5}$, so the requirements of all the types of motes are not satisfied. Similarly, if the portion of motes that use an MCS is chosen in the inverse proportion of the frame duration at the considered MCS, as proposed in [11], then we obtain the average $PLR\approx 1.1\times 10^{-5}$, so the PLR goes down, but remains higher than required.

It is still possible to assign the SFs to the motes in such a way, that the requirements on PLR are satisfied for all types of motes. Let us show how to do it.

**Figure 9:**
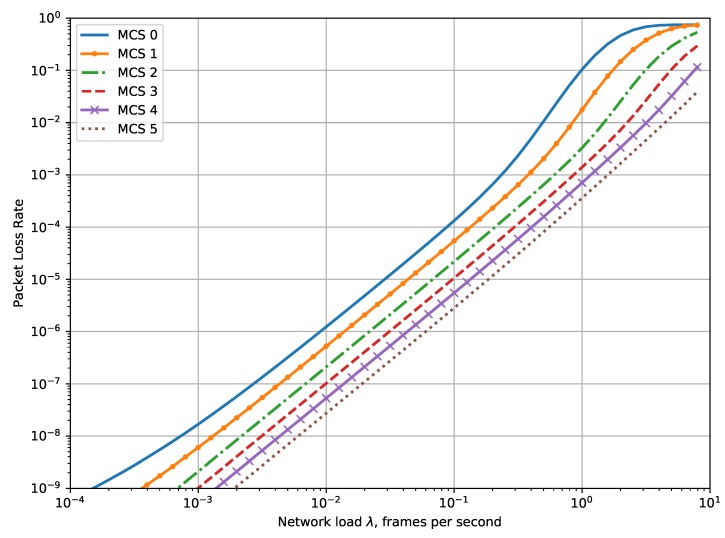
Dependency of PLR on the network load for various MCSs, CR=6 dB.

Figure 9 shows the dependency of PLR on the traffic intensity in the case when all the motes obtain the same MCS. As one can see, a slower MCS provides a higher PLR, and the slowest and the fastest MCS differ in the provided PLRs by two orders of magnitude.

For each mote type and each MCS, we can find the network capacity, i.e., the maximal load which yields the PLR equal to the $PLR^{QoS}$ restriction. Note that the network capacity for some MCS depends on both the traffic at the selected MCS and the *cumulative traffic at all other* MCSs, see (5). Such values characterize the network capacity per each MCS and are shown in Table 2.

Let us assign the MCSs according to the following procedure.

We start with the motes which require the lowest $PLR^{QoS}$, i.e., the motes of type 3. Let us assign MCSs 0 and 1 to the maximal possible number of motes of this type such that the requirement on PLR holds for them. It means that such a number of motes will use these MCSs, that their cumulative load does not exceed the network capacity for the given MCSs, i.e., 0.0006 and 0.0011 frames per second for MCS 0 and MCS 1, respectively.

The remaining motes that generate 0.002−0.00061−0.0011=0.00029 frames per second are assigned to MCS 2. Not the whole capacity has been used for this MCS by far: $1.8\times 10^{-3}-2.9\times 10^{-4}=1.51\times 10^{-4}$ frames per second is the remaining capacity.

Then we consider the next type of motes, namely, type 2. As the entire capacity of MCS 2 has not been used yet, a part of these motes can be assigned this MCS. The part is chosen in such a way that they generate not more than $1.51\times 10^{-4}$ frames per second.

Although for type 2 and MCS 2, the network capacity is much higher, they cannot be further assigned to this MCS, because it results in a violation of the $PLR^{QoS}$ restriction for the type 3 motes that are already assigned to this MCS. For that reason, let us also use the MCS 3 for the type 2 motes: the capacity of this MCS allows us to do it.

The remaining capacity of MCSs 3, 4, and 5 is sufficient to serve the motes of type 1 in such a way that their $PLR^{QoS}$ is satisfied. We can assign MCSs according to the procedure we used for type 2 and 3 motes or can split them evenly between the MCSs 4 and 5 since each MCS allows serving the traffic load of $10^{-1}$ frames per second.

Let us also consider another scenario, when the motes of type 3 generate 5 times more heavy traffic, namely 0.01 frames per second. In such a case, even if we assigned MCSs 0, 1, 2 and 3 to the motes of the third category, then there would remain motes which have not been assigned to an MCS, so we would have to assign the MCS 4 to these motes. The remaining capacity for MCS 4 would be $1.2\times 10^{-4}$, which, together with the capacity for MCS 5 would not suffice to satisfy the requirement on PLR for the motes of types 1 and 2.

Thus with the developed model, we can assign the MCSs to various groups of motes to satisfy the QoS requirements or to determine that the QoS requirements cannot be satisfied.

In our research, we considered that the MCSs are orthogonal, and their assignment can be performed separately from each other. In the studied scenario, such an assumption is justified by the small network radius, which makes it impossible to the signals of different motes to have a huge power difference required to break the orthogonality between different SFs. Also, in the considered scenario, the traffic is rather low, while the impact for inter-SF interference is crucial for high network load, close to 100%. In a bigger network, it might be essential to consider the influence of different MCSs at each other, but a bigger network means that the motes cannot be assigned any MCSs since some MCSs can become unusable due to the distance to the GW. QoS provision in big networks is still a problem for future research.

## 7. Conclusions

In the paper, we considered LoRaWAN networks with class A motes operating in the acknowledged mode.

We have developed an accurate mathematical model of data transmission in LoRaWAN networks. In contrast to previous works, our model allows estimating such important performance indices as the maximal affordable load, packet loss ratio, and delivery time distribution. Our model can be used for performance evaluation, network planning, and MCS selection.

Existing approaches of MCS allocation fail in a heterogeneous network where the motes transmit various types of traffic with different QoS requirements. At the same time, with the developed model, we can allocate MCSs in such a way that QoS requirements are satisfied.

In the future, we are going to improve our model to find not only the average PLR in the network but also the PLR distribution, because this performance indicator may be important in some heterogeneous scenarios when it is crucial to guarantee some minimum level of PLR. We are also planning to consider less compact scenarios, in which the non-orthogonality of SFs cannot be neglected. In such scenarios, the set of MCSs available to a mote can be limited by its distance from the GW, and the allocation should be performed taking into account the interference from the motes that use another MCSs. Another important issue that should be taken into account is the limited number of demodulator paths at the GW [21], which can become important at high network loads.

## Figures and Tables

**Table 1:** Modulation and coding schemes (MCSs) in EU 863-780 MHz ISM band.

#	Spreading Factor	Channel Width, kHz	Code Rate	PHY bit Rate, bps	RF Sensitivity, dBm
0	12	125	4/6	250	−137
1	11	125	4/6	440	−136
2	10	125	4/5	980	−134
3	9	125	4/5	1760	−131
4	8	125	4/5	3125	−128
5	7	125	4/5	5470	−125
6	7	250	4/5	11,000	−122

**Table 2:** Network capacity, frame per second, for various mote types and modulation and coding schemes (MCSs).

MCS	Network Capacity for Type 1	Network Capacity for Type 2	Network Capacity for Type 3
	${\mathrm{PLR}}^{\mathrm{QoS}}=10^{-5}$	${\mathrm{PLR}}^{\mathrm{QoS}}=10^{-6}$	${\mathrm{PLR}}^{\mathrm{QoS}}=10^{-8}$
0	0.025	0.0076	0.00061
1	0.038	0.012	0.0011
2	0.059	0.018	0.0018
3	0.084	0.026	0.0026
4	0.12	0.036	0.0036
5	0.16	0.05	0.005
